# Anomalous aortic origin of the right coronary artery with functional ischemia determined with fractional flow reserve derived from computed tomography

**DOI:** 10.1002/ccr3.1582

**Published:** 2018-05-15

**Authors:** Takashi Miki, Toru Miyoshi, Atsuyuki Watanabe, Kazuhiro Osawa, Naofumi Amioka, Hiroshi Ito

**Affiliations:** ^1^ Department of Cardiovascular Medicine Okayama University Graduate School of Medicine, Dentistry and Pharmaceutical Sciences Okayama Japan

**Keywords:** computed tomography, coronary artery anomaly, fractional flow reserve, ischemia

## Abstract

A right coronary artery of anomalous origin is rare congenital anatomy that can be fatal. CT angiography is an excellent tool for its anatomical assessment. Noninvasive CT‐based fractional flow reserve measurement can additionally evaluate the functional severity of coronary stenosis and is potentially useful for evaluating coronary anomalies.

## 1 CASE REPORT

**Figure 1:**
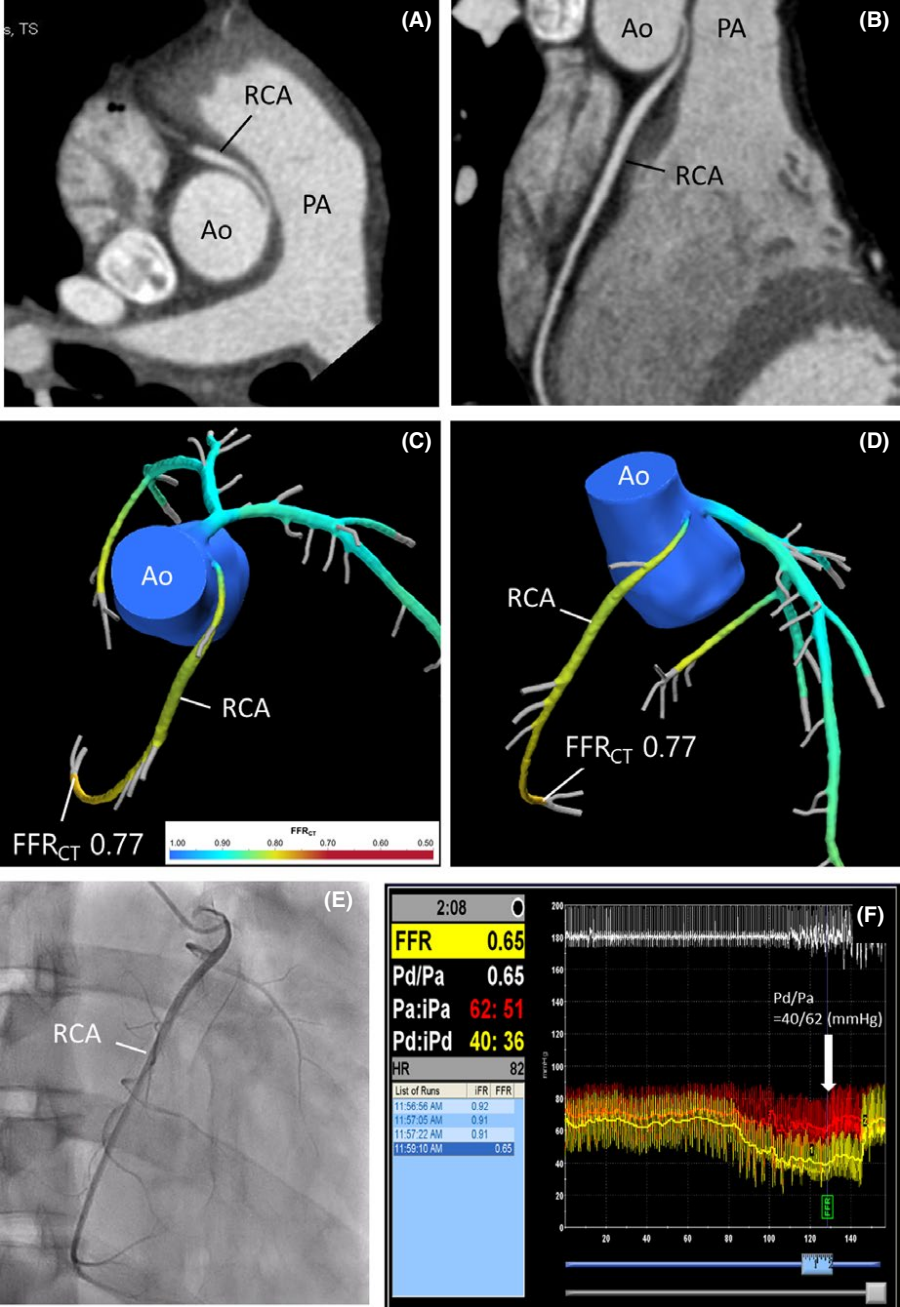
A‐B, Axial and sagittal coronary CT angiography. C‐D, FFR_CT_. E, Coronary angiography, right anterior oblique projection. F, Invasive FFR [= distal coronary/aortic pressures ratio (Pd/Pa)] measured with adenosine 140 μg/kg/min i.v. administration (arrowhead). Ao, aorta; FFR_CT_, CT‐derived fractional flow reserve; PA, pulmonary artery; RCA, right coronary artery

A 26‐year‐old man who was referred to our hospital presented with a clinical history of exercise‐related syncope. Coronary CT angiography revealed an anomalous aortic origin of the right coronary artery (RCA) from the left coronary ostium. The proximal portion of the RCA seemed to be coursing along the aortic vessel wall before running between the aorta and pulmonary artery (Figure 1A,B). CT‐based fractional flow reserve (FFR_CT_) (HeartFlow, Redwood, CA, USA) in the RCA was 0.77 (ischemic cutoff ≤0.8), suggesting significant ischemia (Figure 1C,D). Coronary angiography suggested the presence of a slit‐like ostium of the anomalous RCA (Figure 1E). Invasive FFR evaluation confirmed significant ischemia (Figure 1F). The treating physicians decided on surgical treatment.

An anomalous origin of the RCA from the left sinus of Valsalva is rarely seen congenital anatomy. Although the presentation is usually silent, clinical manifestations may include aborted sudden death, chest pain, arrhythmia, and/or exercise‐induced presyncope or syncope. The FFR_CT_, derived from the usual data set from coronary CT angiography, was recently developed to evaluate functional ischemia of the coronary artery.^1^ In the present case, FFR_CT_ revealed ischemia in an anomalous coronary artery, which was confirmed by the invasive FFR measurement. Thus, the FFR_CT_ technique has potential for innovation in the assessment of anomalous coronary arteries.

## AUTHORSHIP

All authors made substantial contributions to the preparation of this manuscript. TMiy, TMik, KO, NA, and AW: involved in the conceptualization, preparation, writing, and review of this manuscript. HI: involved in the conceptualization, writing, and review of this manuscript.

## CONFLICT OF INTEREST

The authors declare that there is no conflict of interest regarding the publication of this article.
